# Markers of achievement for assessing and monitoring gender equity in a UK National Institute for Health Research Biomedical Research Centre: A two-factor model

**DOI:** 10.1371/journal.pone.0239589

**Published:** 2020-10-14

**Authors:** Lorna R. Henderson, Syed Ghulam Sarwar Shah, Pavel V. Ovseiko, Rinita Dam, Alastair M. Buchan, Helen McShane, Vasiliki Kiparoglou

**Affiliations:** 1 National Institute for Health Research Oxford Biomedical Research Centre, John Radcliffe Hospital, Oxford, United Kingdom; 2 Radcliffe Department of Medicine, University of Oxford, Oxford, Oxford, United Kingdom; 3 Nuffield Department of Medicine, University of Oxford, Oxford, Oxford, United Kingdom; 4 Nuffield Department of Primary Health Care Sciences University of Oxford, Oxford, United Kingdom; Aalborg University, DENMARK

## Abstract

**Background:**

The underrepresentation of women in academic medicine at senior level and in leadership positions is well documented. Biomedical Research Centres (BRC), partnerships between leading National Health Service (NHS) organisations and universities, conduct world class translational research funded by the National Institute for Health Research (NIHR) in the UK. Since 2011 BRCs are required to demonstrate significant progress in gender equity (GE) to be eligible to apply for funding. However, the evidence base for monitoring GE specifically in BRC settings is underdeveloped. This is the first survey tool designed to rank and identify new GE markers specific to the NIHR BRCs.

**Methods:**

An online survey distributed to senior leadership, clinical and non-clinical researchers, trainees, administrative and other professionals affiliated to the NIHR Oxford BRC (N = 683). Participants ranked 13 markers of GE on a five point Likert scale by importance. Data were summarised using frequencies and descriptive statistics. Interrelationships between markers and underlying latent dimensions (factors) were determined by exploratory and confirmatory factor analyses.

**Results:**

The response rate was 36% (243 respondents). Respondents were more frequently female (55%, n = 133), aged 41–50 years (33%, n = 81), investigators (33%, n = 81) affiliated to the BRC for 2–7 years (39.5%, n = 96). Overall participants ranked ‘BRC senior leadership roles’ and ‘organisational policies on gender equity’, to be the most important markers of GE. 58% (n = 141) and 57% (n = 139) respectively. Female participants ranked ‘organisational policies’ (64.7%, n = 86/133) and ‘recruitment and retention’ (60.9%, n = 81/133) most highly, whereas male participants ranked ‘leadership development’ (52.1%, n = 50/96) and ‘BRC senior leadership roles’ (50%, n = 48/96) as most important. Factor analyses identified two distinct latent dimensions: “organisational markers” and “individual markers” of GE in BRCs.

**Conclusions:**

A two-factor model of markers of achievement for GE with “organisational” and “individual” dimensions was identified. Implementation and sustainability of gender equity requires commitment at senior leadership and organisational policy level.

## Introduction

Underutilisation of female talent and potential in academic medicine, particularly at senior levels and leadership roles, as well as the health workforce more broadly is well documented [1–4]. This has been referred to as a “leaky talent pipeline” [5].

In 2011 the challenge to address gender equity (GE) in medical schools was linked directly to BRCs in England. The UK Department of Health’s Chief Medical Officer announced that (NIHR) would not shortlist any National Health Service (NHS) / University partnership for NIHR BRC designation and funding: “where the academic partner (generally the Medical School/Faculty of Medicine) has not achieved at least a Silver Award of the Athena SWAN Charter for Women in Science” [6].

### Athena SWAN charter

The Athena SWAN charter advances women’s careers in Universities in terms of representation, progression of students into academia, journey through career milestones and working environment [7]. Universities may be awarded Bronze, Silver or Gold Athena SWAN award, based on their action plans, achievements and impact in advancing gender equity [7]. Athena SWAN awards are useful markers of GE achievement in Universities but not specifically designed for translational research organisations (TROs) such as NIHR BRCs, partnerships between UK's leading NHS organisations and universities [8]. Furthermore, recent GE research has focussed on Universities [9–13]. There is therefore a gap in GE research and practice in the context of NIHR BRCs [14]. This study aims to address gap by identifying new markers of achievement for assessing and monitoring GE in NIHR BRCs.

## Methods

### Study aims and objectives

The aims of this study are two fold: firstly, to inform women’s advancement in translational research settings through the development of markers of achievement for assessing and monitoring gender equity. Secondly, to test and develop a survey tool which captures the major dimensions of gender equity in the NIHR BRC to inform future planning and monitoring of GE in a Biomedical research setting [14].

We adopt the UNESCO definition of gender equity: “fairness of treatment for women and men, according to their respective needs. This may include equal treatment or treatment that is different but which is considered equivalent in terms of rights, benefits, obligations and opportunities” [15].

### Study setting

The NIHR is the UK’s largest funder of health and care research [16]. There are currently 20 BRCs: collaborations between universities and NHS organisations bringing together academics and clinicians to translate scientific breakthroughs into new treatments, diagnostics and medical technologies [17]. The study was conducted at the NIHR Oxford BRC—a collaboration between the Oxford University Hospitals NHS Foundation Trust and the University of Oxford. It is based at the Oxford University Hospitals—one of the largest UK acute teaching hospitals with an International reputation for services and research [18]. It is run in partnership with the University of Oxford which is consistently ranked as the world’s best institution for medical teaching and research [19].

The NIHR Oxford BRC was established in 2007 with competitive funding from NIHR of £57 million over 5 years, £96 million in 2012 to recognise its outstanding contribution to research and £113.6 million in 2016 making it one of the largest BRCs in the UK [18]. This funding supports NHS clinicians and world leading academics to conduct translational research. The BRC is divided into twenty research themes (e.g. Genomics, Cardiovascular, Diabetes, etc.) and four clusters (Precision Medicine, Technology and Big Data, Immunity and Infection and Chronic Diseases) [18].

### Study population

In contrast to existing studies focussing on GE in Universities, our study population is intentionally broader including both university and NHS employees.

Study population (N = 683) including all researchers and affiliates were invited to participate. The participants were categorised as Investigators: researchers leading and undertaking research, associates supporting research led by others (i.e. facilitators and administrative staff), and academic trainees (trainees/PhD students). In addition, patient and public involvement representatives, industry managers and leaders (including senior executive and non-executive committees) funded / supported the NIHR Oxford BRC (herewith referred to as BRC affiliates) were invited to participate. Names and contact details of all affiliates were extracted from the BRC’s internal databases. To ensure accuracy, all BRC theme managers were also contacted and asked to provide up to date email addresses of affiliates within their respective themes. List of participants invited to survey included 311 male names (45.5%) and 372 female names (54.5%). There were 341 (49.9%) investigators ((e.g. PI (Principal Investigators) / co-PI / CI (co-investigators)), 210 (30.7%) research associates (e.g. researchers and research fellows), 25 (3.7%) trainees/PhD students, 79 (11.6%) administrative / technical and other professional staff, and 28 (4.1%) other professionals associated with the BRC.

### Development of the questionnaire

Participants were asked to rank the importance of 13 markers of achievement of GE in BRCs on a five point Likert scale: Very important” (score 5), “Important” (score 4), “Neutral” (score 3), “Not important” (score 2) and “Not at all important” (score 1). Potential markers were identified from the literature reported in the study protocol [14]. We then checked the face validity of the identified potential markers.

Participants’ were asked to provide demographic characteristics i.e. age, gender; current role in the NIHR Oxford BRC and how long they had been affiliated to the BRC. Taking into account the diverse identities of women and men and based on the University of Oxford staff survey categories we did not use a binary sex indicator for gender but added “self describe” and “not report” following guidance from the University of Oxford’s equality and diversity team.

### Piloting of the questionnaire

The questionnaire was piloted in face-to-face interviews with potential participants (n = 10) to ensure it was easily understood and met the purpose of what it was intended to measure. It was also tested via email to a small sample (n = 16) from the population of interest to assess readability and clarity of the items and appropriateness of participants interpretations. Following piloting, a few minor changes were made in wording and formatting prior to the main survey study (S1 Appendix).

### Administration of survey

The survey was conducted from May to July 2019. The NIHR Oxford BRC’s Chief Operating Officer sent an email via SurveyMonkey® with a web link to the anonymous online survey to all survey participants (N = 683) informing them about the survey. The clinical research manager of the NIHR Oxford BRC also sent an email to BRC theme liaisons (theme managers) to inform their theme members, i.e. theme leaders, researchers and supporting BRC affiliates about the survey. Up to 3 automated email reminders over the 6 weeks were sent via SurveyMonkey® to participants who had not completed or partially completed the survey.

### Data analysis

Online data from SurveyMonkey® was downloaded in SPSS and Microsoft Excel spreadsheet formats. Frequencies of participants’ demographic characteristics and descriptive statistics of scores of the importance of 13 markers of GE in BRCs were analysed. The Mann-Whitney U test was used to determine statistical differences in ranking the importance of GE markers by participants’ gender (only male and female categories). The Kruskall-Wallis H test with Bonferroni corrections was applied to evaluate differences in ranking markers by participants’ age (3 categories: 18–40 years, 41–50 year, and 51 and more years), BRC role (3 categories: Investigators, research associates, and admin/tech/prof. staff) and duration of affiliation to the BRC (3 categories: up to 2 years, 3–7 years, and more than 7 years). For statistical significance, a p-value <0.05 was applied.

Thereafter, data on participants’ scores of the importance of 13 markers of GE in BRCs were analysed using exploratory and confirmatory factor analyses for identifying underlying latent factors / constructs, as described below.

#### Exploratory factor analysis

We determined interrelationships between markers and underlying latent dimensions (factors) by exploratory factor analysis (EFA) [20]. EFA was run to extract the latent factors (dimensions) covered in the measured 13 markers of GE. For the EFA, we used Principal Component Analysis (PCA) as a factor-extraction method, the Varimax with Kaiser Normalization as a rotation method and the Kaiser’s Eigen values > 1 (EVG1) criterion and breaks in the scree plot for determining the number of latent factors [21]. We applied minimum communalities ≥0.50, with no cross loadings ≥0.45 on more than one latent factor [21] and the minimum acceptable factor loading as 0.50 on only one factor [20]. Our sample size was 243 and the participant-to-variable ratio was 18:1, which was higher than the minimum acceptable participant-to-variable ratio of 10:1 [20].

#### Confirmatory factor analysis

Subsequent to the EFA, we ran the confirmatory factor analysis (CFA) [22]. The internal consistency of latent dimensions identified in the EFA was checked by running scale reliabilities using the Cronbach’s alpha coefficient [23]. The measurement model identified in CFA was checked for convergent and discriminant validity by calculating the Average Variance Extracted (AVE) as suggested [20, 24].

Participants’ ratings of GE markers were positively skewed, which was reduced by log transformation prior to running EFA and CFA [22]. All statistical analyses were undertaken using IBM SPSS Statistics for Windows, version 25.0 (IBM Corp INC: Armonk, NY) except the CFA for which we used the IBM SPSS AMOS for windows, version 26.0 (IBM Corp INC: Armonk, NY).

### Ethics

The study was reviewed by Oxford University Medical Sciences Inter-divisional Research Ethics Committee and University of Oxford Clinical Trials and Research Governance office who determined that the study was exempt from full ethical review. The information sheet provided on the first page of the online survey informed participants that their participation in the survey was voluntary and they could withdraw at any time. They were also informed that their data and responses provided in the survey would be held securely, confidential, processed and reported anonymously and in aggregated format. Participants were informed that ‘if you do not wish to complete the survey, please click on ‘No, I do not consent’ and then the survey will be aborted’. Consequently, only those participants who gave their online informed consent by clicking the option ‘Yes, I consent’ were able to complete the survey via SurveyMonkey®.

## Results

### Response rate

The survey was completed by 277 out of 683 participants invited. 34 responses were ineligible for inclusion as they provided partial or missing data; hence, were removed from the sample and data analysis. One participant did not consent and opted out of the survey. Therefore, the final sample comprised 243 respondents and the effective response rate was 36%.

### Demographic characteristics of participants

The majority of respondents were female (55%, n = 133), aged 41–50 years (33.3%, n = 81), investigators e.g. principal, co and chief investigators (33.3%, n = 81) affiliated with the BRC for 2–7 years (39.5%, n = 96) (Table 1).

**Table 1:** Socio-demographic characteristics of survey respondents.

Socio-demographic characteristics	Frequency	Percentage
Gender		
Female	133	54.7
Male	96	39.5
Prefer to self describe	3	1.2
Prefer not to say	10	4.1
Missing data	1	0.4
Age (years)		
18–30	21	8.6
31–40	60	27.4
41–50	81	33.3
51–60	52	21.4
61+	17	7.0
Prefer not to say	11	4.5
Missing data	1	0.4
Affiliate category / Role in the BRC		
Investigators (e.g. PI/co-PI/CI)	81	33.3
Research Associates (e.g. Researchers and research fellows)	67	27.6
Admin/technical/Professional/Support Associates	59	24.3
Trainees/PhD students	3	1.2
Other	21	8.6
Prefer not to say	9	3.7
Missing data	3	1.2
Duration of affiliation with the BRC		
Up to 2 years	86	35.4
3-7years	96	39.5
More than 7 years	52	21.4
Prefer not to say	6	2.5
Missing data	3	1.2

### Ranking of markers of achievement for gender equity

Table 2 presents participants’ rankings of the importance of 13 markers of GE. The top two markers with the highest overall mean rankings were BRC senior leadership roles (mean = 4.43, standard deviation (SD) = 0.80) and organisational policies on gender equity (mean = 4.40, SD = 0.85).

**Table 2:** Participants ranking* of markers of achievement of gender equity in biomedical research centres (N = 243).

	Descriptive statistics (rating by all participants)							Differences in rating by gender^a^—Male and Female participants only				
	Mean	Standard Deviation	Median	Mode	Percentiles			Gender		Mann-Whitney U	Z score	P Value (2-tailed)
Markers of achievement of gender equity					25	50	75	Male	Female			
1. BRC senior leadership roles: e.g. Director, Steering Committee Member, Theme leader & Co-lead.	4.43	0.8	5	5	4	5	5	4.35	4.53	5492	-2.055	0.04
2. Leadership development: e.g. Gender-sensitive leadership programmes, succession plans.	4.34	0.91	5	5	4	5	5	4.39	4.40	6128	-0.582	0.561
3. BRC staff category: e.g. Principal Investigator, Researchers, Trainees and Admin & Support staff.	4.33	0.87	5	5	4	5	5	4.26	4.46	5471	-2.053	0.04
4. Recruitment & retention: e.g. Number of Staff recruited and promoted.	4.34	0.86	5	5	4	5	5	4.24	4.50	5211	-2.651	0.008
5. BRC funding: e.g. Distribution by Theme, Gender and Role.	4.13	0.96	4	5	4	4	5	4.03	4.28	5335	-2.278	0.023
6. External grant funding: e.g. Total amount, Role on the grant, Number of grants and Success rate.	4.09	0.94	4	5	4	4	5	4.01	4.24	5444	-2.032	0.042
7. Esteem indicators: e.g. NIHR Senior Investigators, Funding panel membership, Invited plenary speakers, Fellowships of learned societies, Honours and Awards.	4.25	0.91	4	5	4	4	5	4.20	4.38	5522	-1.912	0.056
8. Publications: e.g. Authorship (First / Corresponding / Senior author) and Type of Publication (Journal articles and Conference papers).	4.09	0.97	4	5	4	4	5	4.02	4.21	5483	-1.955	0.051
9. Intellectual property: e.g. Number of Patents, Licenses and Spinouts.	3.9	1.06	4	5	3	4	5	3.80	4.05	5505.5	-1.867	0.062
10. Collaboration with industry: e.g. Board membership, Joint grants and Advisory roles (non-executive directorships).	3.99	1	4	4	3	4	5	3.91	4.13	5434	-2.045	0.041
11. Patient and public involvement: e.g. Representative Number of Men and Women Speakers and Participants.	4.19	0.9	4	5	4	4	5	4.14	4.31	5536	-1.856	0.063
12. Organisational policies on gender equity: e.g. Personal Development Training, Mentoring, Sponsorship and Career Development.	4.4	0.85	5	5	4	5	5	4.35	4.49	5462	-2.115	0.034
13. Organisational Targets: e.g. Creating BRC targets for Gender Equity.	4.21	0.98	4	5	4	4	5	4.14	4.36	5375	-2.23	0.026

*Scores: 1 = Not important at all, 2 = Not important, 3 = Neutral, 4 = Important, 5 = Very Important. a. Grouping Variable: Gender—male and female only.

**Fig 1:**
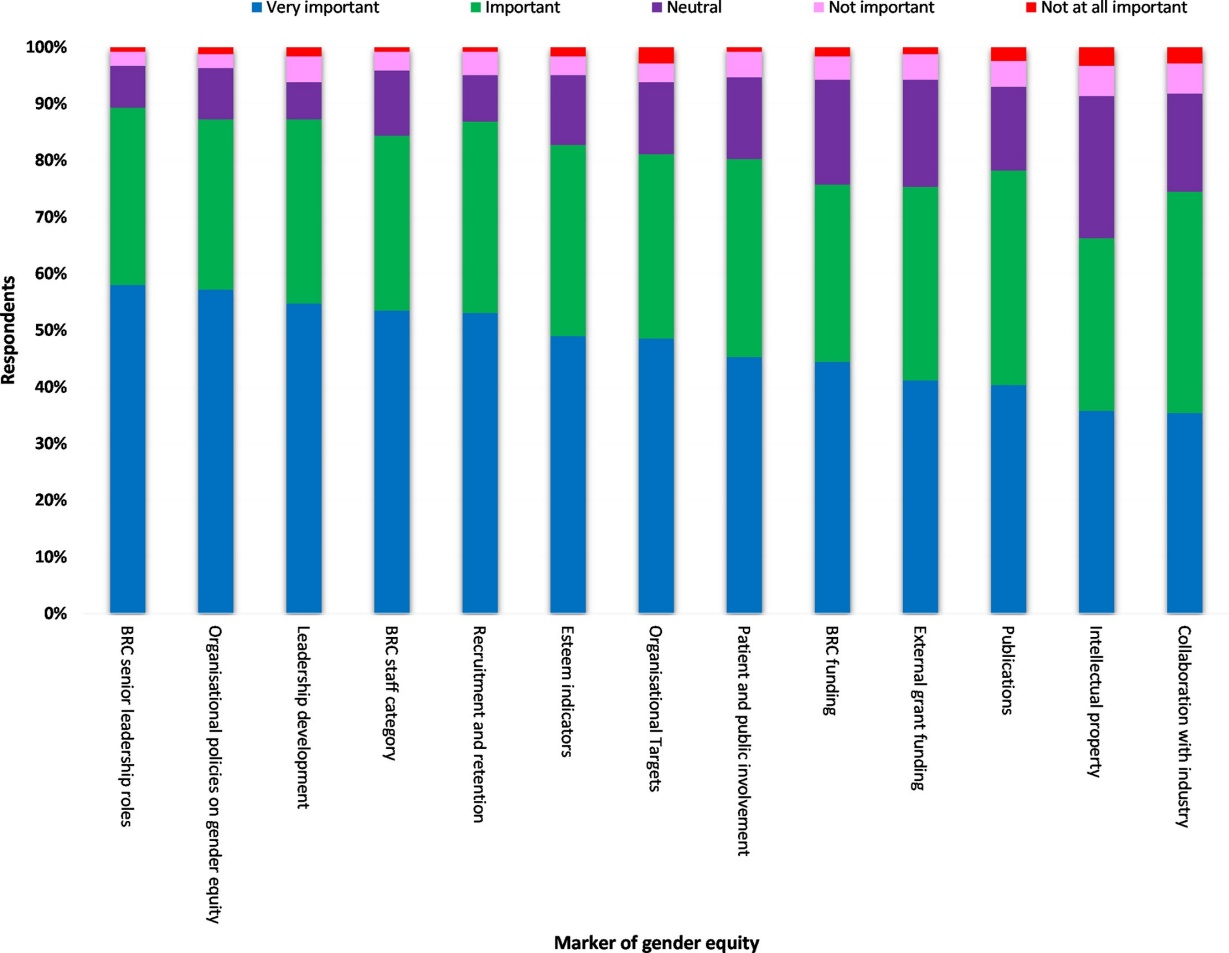
Importance of markers of gender equity in BRCs by all respondents.

When participants scores were combined, the majority (58%, n = 141) scored BRC senior leadership roles as a very important marker of GE. Organisational policies on GE ranked as the second highest very important marker by 57.2% (n = 139) of participants (Fig 1). Collaboration with industry and Intellectual property emerged as the last and second last very important markers of GE in BRCs 35.4% (n = 86) and 35.8% (n = 87) respectively (Fig 1).

**Fig 2:**
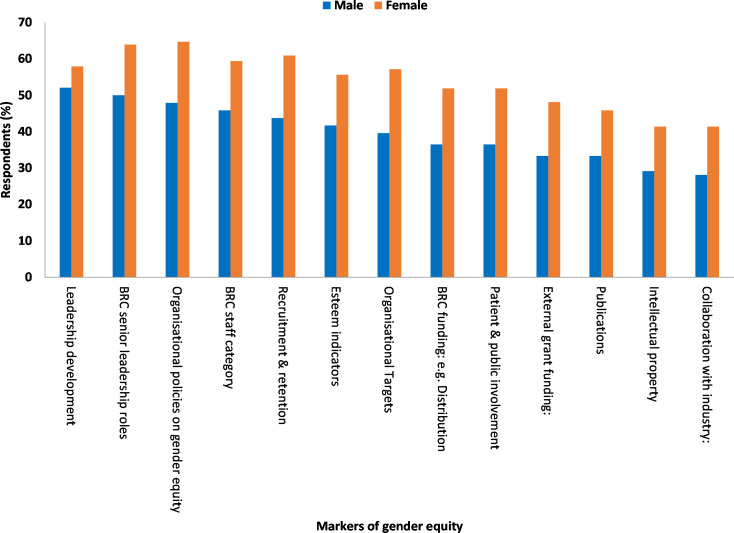
Markers of gender equity marked as very important by gender.

The Mann-Whitney U test results showed that statistically significant differences in the mean rankings by gender i.e. male and female participants for eight markers. These were BRC senior leadership roles (U = 5492 p = 0.040), BRC staff category (U = 5471, p = 0.040), recruitment and retention, (U = 5211, p = 0.008), BRC funding (U = 5335, p = 0.023), external grant funding, (U = 5444, p = 0.042), collaboration with industry (U = 5434, p = 0.041), organisational policies on gender equity (U = 5462, p 0.034), and organisational targets (U = 5375, p = 0.026 (Table 2). Overall, a higher proportion of female participants ranked all 13 markers of GE as the most important marker compared to male participants (Fig 2).

We created a priority ranking order from 1 to 13 of all markers of GE ((highest (Rank 1) lowest (Rank 13)) based on the percentage of participants ranking each marker as very important (Table 3).

**Table 3:** Priority ranking order of markers of gender equity by participants’ gender, role in the BRC, duration of affiliation with the BRC and age.

	Very important scores only																									
	Gender				Role in the BRC						Duration of affiliation to the BRC						Age group									
	Male		Female		Investigators		Research associates		Admin/Tech/Prof. Staff		Up to 2 years		3–7 years		>7 years		18–30 years		30–40 years		41–50 years		51–60 years		60+ years	
Markers of gender equity	Rank	%	Rank	%	Rank	%	Rank	%	Rank	%	Rank	%	Rank	%	Rank	%	Rank	%	Rank	%	Rank	%	Rank	%	Rank	%
1. Leadership development	1	53.7	5	60.6	1	65.7	6	59	4	44.2	4	53.9	3	57.5	2	64.9	6	43.8	6	60.8	1	50.8	1	62.5	1	78.6
2. BRC senior leadership roles	2	52.4	3	64.4	2	64.3	2	65.6	3	44.2	2	56.6	2	57.5	1	67.6	2	56.3	3	64.7	2	49.2	2	62.5	2	78.6
3. BRC Staff category	3	47.6	4	61.5	3	60	3	65.6	7	38.5	5	53.9	4	54.8	4	59.5	7	43.8	4	62.7	4	46.2	4	57.5	3	78.6
4. Recruitment and retention	5	45.1	2	65.4	4	58.6	1	68.9	5	40.4	3	56.6	5	53.4	5	59.5	4	50	1	72.5	5	43.1	5	57.5	5	64.3
5. BRC funding	8	36.6	8	53.8	5	52.9	8	50.8	9	30.8	6	53.9	8	46.6	10	51.4	12	37.5	8	51	8	40	8	52.5	11	50
6. Esteem indicators	6	42.7	7	57.7	6	52.9	5	60.7	8	34.6	9	44.7	6	52.1	3	62.2	3	56.3	7	58.8	7	40	9	50	4	71.4
7. Organisational policies on gender equity	4	47.6	1	66.3	7	52.9	4	65.6	1	53.8	1	57.9	1	60.3	6	54.1	1	75	2	64.7	3	47.7	3	60	6	57.1
8. Organisational Targets	7	40.2	6	58.7	8	47.1	7	57.4	2	46.2	8	48.7	7	50.7	7	54.1	8	43.8	5	62.7	6	40	7	52.5	8	57.1
9. External grant funding	10	32.9	10	49	9	45.7	11	47.5	11	28.8	7	53.9	9	45.2	8	54.1	9	43.8	10	41.2	9	35.4	10	50	12	50
10. Publications	11	32.9	11	45.2	10	42.9	9	50.8	13	23.1	11	31.6	11	41.1	9	54.1	10	43.8	11	37.3	10	35.4	11	45	13	50
11. Patient and public involvement	9	35.4	9	51.9	11	41.4	10	50.8	6	40.4	10	43.4	10	42.5	11	51.4	11	43.8	9	49	11	30.8	6	57.5	7	57.1
12. Intellectual property	12	30.5	13	41.3	12	38.6	12	44.3	12	25	13	27.6	12	39.7	13	48.6	13	37.5	12	33.3	12	30.8	13	42.5	10	57.1
13. Collaboration with industry	13	29.3	12	42.3	13	35.7	13	41	10	30.8	12	27.6	13	38.4	12	51.4	5	50	13	29.4	13	29.2	12	45	9	57.1

### Differences in ranking by gender

Men ranked “leadership development” (53.7%) and “BRC senior leadership roles”, (52.4%) as the most important markers of GE(53.7% and 52.4% respectively). Conversely, women ranked “organisational policies on gender equity” (66.3%) and “recruitment and retention” (65.4%) (66.3% and 65.4% respectively) (Table 3).

### Differences in ranking by role

Ranking also differed by participants’ seniority. Investigators ranked “Leadership development” to be the most important (65.7%), associates ranked “recruitment and retention” (68.9%) whereas less senior staff ranked “organisational policies on gender equity” for 53.8% as most important (53.8%) (Table 3). The Kruskal-Wallis H test with Bonferroni corrections showed that the mean scores of ranking the importance of the markers by the role in the BRC (3 categories: Investigators, research associates, and admin/tech/prof. staff) were statistically significantly different for six markers: BRC senior leadership roles (χ^2^ (2) = 8.89, p = .012), BRC staff category (χ^2^ (2) = 14.39, p = .001), recruitment and retention (χ^2^ (2) = 13.25, p = .001), BRC funding (χ^2^ (2) = 11.46, p = .003), external grant funding (χ^2^ (2) = 12.12, p = .002), and publications (χ^2^ (2) = 10.59, p = .005) (Table 3).

### Differences in ranking by duration of affiliation to BRC

Participants affiliated to the BRC for over seven years ranked “BRC senior leadership roles” as most important marker (67.6%) in contrast those affiliated for up to 2 years or 3–7 years ranked “organisational policies on gender equity” most highly (57.9% and 60.3% respectively) (Table 3).

### Differences in ranking by age

“Leadership development” was the top most important marker of GE for participants aged 40 years and older whereas the under 40 group ranked notably lower (rank 6). “Organisational policies on gender equity” were the most important marker for a notably high number of younger participants (75% aged 18–30 years), similarly “recruitment and retention” for 72.5% of participants the 30–40 years career group whereas the >40 career group ranked it notably lower (rank 5) (Table 3).

However, Kruskal-Wallis H tests with Bonferroni corrections showed that there were no statistically significant differences in the mean rankings of all markers between different age categories (3 categories: 18–40 years, 41–50 year, and 51 and more years) or duration of affiliation to the BRC (3 categories: up to 2 years, 3–7 years, and more than 7 years).

### Exploratory factor analysis

Results of the first EFA model that included all 13 measured markers revealed a two factor solution but the rotated component matrix showed that marker No. 5 (i.e., BRC funding) had very high cross loadings i.e., 0.53 on factor 1 and 0.68 on factor 2. We removed this marker and re-ran the EFA model with 12 markers, which again resulted in a 2 latent factor solution but again the rotated component matrix revealed that measured marker No. 7 (i.e., esteem indicators) had very high cross loadings i.e., 0.50 and 0.67 on factor 1 and factor 2 respectively. Consequently, we removed marker No. 7 and re-ran the EFA, which again resulted in a 2 latent factor solution with the rotated component matrix showing marker No. 11 (i.e., patient and public involvement) with higher cross loadings i.e., 0.61 on factor 1 and 0.45 on factor 2.

Subsequently, we removed marker No. 11 from the EFA and re-ran the model, which showed no marker having cross loading ≥0.45 on more than one factor “Table 4”. We selected this model as a final EFA model with the Kaiser-Meyer-Olkin (KMO) Measure of Sampling Adequacy = 0.884 and statistically significant Bartlett's Test of Sphericity (χ^2^ = 1553.69, p < 0.0001), which confirmed the suitability of the data for running the EFA model. Table 4 presents the statistics about the extracted communalities, total variance explained and Rotated Factor Matrix. Based on the content of loaded measured items on latent factor 1 and factor 2, we identified these markers as the organisational and individual markers respectively (Table 4).

**Table 4:** Exploratory factor analysis: Measured markers of gender equity, latent factors with loadings, communalities, Eigen values, KMO Measure of Sampling Adequacy, Bartlett's Test of Sphericity, total variance explained and scale reliabilities.

Rotated Component Matrix^a^			
Measured items/ Markers of gender equity	Component loadings		Communalities
	Factor 1 (Organisational markers)	Factor 2 (Individual markers)	*h*^2^
BRC senior leadership roles	**.84**	.30	.80
Leadership development	**.81**	.29	.74
BRC staff category	**.78**	.39	.76
Recruitment & retention	**.77**	.28	.67
External grant funding	.42	**.79**	.79
Publications	.34	**.83**	.80
Intellectual property	.34	**.87**	.87
Collaboration with industry	.30	**.85**	.81
Organisational policies on gender equity	**.68**	.33	.58
Organisational Targets	**.72**	.35	.64
*Eigenvalues*	*6*.*40*	*1*.*06*	
*Kaiser-Meyer-Olkin (KMO) Measure of Sampling Adequacy*	.*884*		
*Bartlett's Test of Sphericity*	*χ2 = 1553*.*69*		
*Significance (P)*	*< 0*.*0001*		
*of total variance explained*	*63*.*98*	*10*.*65*	
*Cronbach’s α reliability (Standardised)*	.*912*	.*925*	

^a^Extraction Method: Principal Component Analysis; Rotation Method: Varimax with Kaiser Normalization; Rotation converged in 3 iterations.

### Confirmatory factor analysis

**Fig 3:**
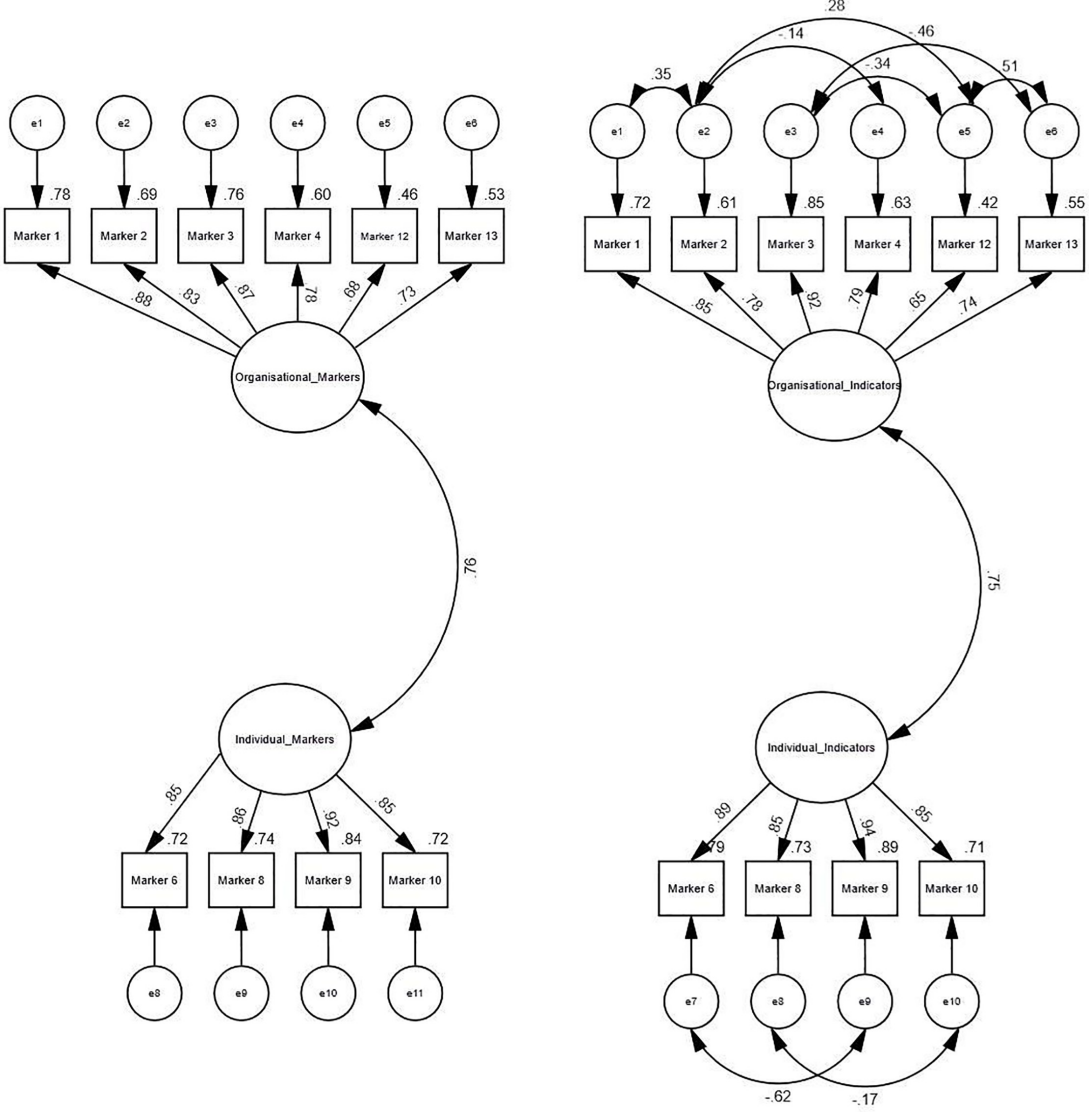
A Initial CFA-model. Notes: Rectangles represent measured items (endogenous variables); circles represent latent (unmeasured/exogenous) variables. Marker 1 (BRC senior leadership roles), Marker 2 (Leadership development), Marker 3 (BRC staff category), Marker 4 (Recruitment and retention), Marker 6 (External grant funding), Marker 8 (Publications e.g. authorship), Marker 9 (Intellectual property), Marker 10 (Collaboration with industry), Marker 12 (Organisational policies on gender equity) and Marker 13 (Organisational Targets for gender equity). B Post-hoc CFA model. Notes: Rectangles represent measured items (endogenous variables); circles represent latent (unmeasured/exogenous) variables. Marker 1 (BRC senior leadership roles), Marker 2 (Leadership development), Marker 3 (BRC staff category), Marker 4 (Recruitment and retention), Marker 6 (External grant funding), Marker 8 (Publications e.g. authorship), Marker 9 (Intellectual property), Marker 10 (Collaboration with industry), Marker 12 (Organisational policies on gender equity) and Marker 13 (Organisational Targets for gender equity).

To check the two latent factor solution observed in the EFA (Table 4), we ran CFA (henceforth mentioned as the initial CFA model) as shown in Fig 3A.

A model summary of the goodness of fit (GoF) indices for the initial CFA model is presented in (Table 5). The GOF indices suggested that the initial CFA model did not fit with the data. Consequently we created post-hoc modifications by correlating error estimates of some parameters (measured items) as suggested in the Modification indices in the initial AMOS model (Fig 3A). This helped in improving the model fit to given data in the final model (Fig 3B), henceforth mentioned as the post-hoc CFA model.

**Table 5:** Summary of goodness of fit indices observed in the initial and post-hoc CFA models.

Values	χ^2^	Df	Sig (*p*)	χ^2^/df	IFI	NFI	CFI	RMSEA
Recommended			>0.05	≤3.00	≥0.9	≥0.9	≥0.9	≤0.08 (p>0.05)
Observed in initial CFA model	239.95	43	<0.0001	5.58	0.88	0.86	0.88	0.16‡
Observed in Post-hoc CFA model	48.81	26	0.004	1.88	0.98	0.97	0.99	.07‡(Low 90.038, High 90.098), p Close 0.141

χ^2^, Chi-square; Df, degrees of freedom; Sig, significance level (p),CFI, comparative fit index; IFI, Incremental fit index; NFI, normed fit index; RMSEA, root mean square error of approximation

‡90 confidence interval for RMSEA.

The GOF indices of the post-hoc model (Table 5) showed a good fit of the post-hoc model with the data. We therefore accepted the post-hoc CFA model as the final CFA model.

The estimates of standardized regression weights (β) of measured items on to the latent factors along with their significance level (p) observed in both the initial and the post-hoc CFA models (Table 6), demonstrate that all measured markers had statistically significant higher loadings on organisational makers factor (β ≥0.68, p<0.001) and individual markers factor (β ≥0.85, p<0.001) (Table 6, Fig 3A and 3B).

**Table 6:** Latent factors, measured markers and standardised estimates observed in initial and post-hoc CFA models.

			Initial CFA Model			Post-hoc CFA Model		
Latent factors		Measured markers	Estimate† (β)	C.R.	P	Estimate† (β)	C.R.	P
Organisational Markers	→	Marker 1	0.88	‡	‡	0.85	‡	‡
	→	Marker 2	0.83	15	***	0.78	15.9	***
	→	Marker 3	0.87	16.4	***	0.92	16.1	***
	→	Marker 4	0.78	13.3	***	0.79	13.2	***
	→	Marker 12	0.68	10.7	***	0.65	9.34	***
	→	Marker 13	0.73	12.1	***	0.74	10.3	***
Individual Markers	→	Marker 10	0.85	‡	‡	0.85	‡	‡
	→	Marker 9	0.92	16.7	***	0.94	16.1	***
	→	Marker 8	0.86	15.1	***	0.86	14.1	***
	→	Marker 6	0.85	14.7	***	0.89	14.5	***

Marker 1 (BRC senior leadership roles), Marker 2 (Leadership development), Marker 3 (BRC staff category), Marker 4 (Recruitment and retention), Marker 6 (External grant funding), Marker 8 (Publications e.g. authorship), Marker 9 (Intellectual property), Marker 10 (Collaboration with industry), Marker 12 (Organisational policies on gender equity) and Marker 13 (Organisational Targets for gender equity)

†Estimates of standardized regression weights

‡Not estimated because of loading set to fixed value i.e., 1.0

*** Significance value (p): < .001.

We checked the internal consistency and convergence of both latent factors by the composite reliability and average variance extracted (AVE) respectively. Six measured items (markers) that loaded on to the organisational markers factor explained 64 AVE and 63 AVE in the initial and the post-hoc CFA models respectively whereas four items that loaded on to the individual markers factor explained 76 AVE and 78 AVE in the initial and post-hoc CFA models respectively. The observed AVE for both factors was higher than the minimum 0.5 that is suggested for adequate convergence [17, 18].

We calculated the composite reliability for the organisational markers factor as 0.91 for both the initial and the post-hoc CFA models and the composite reliability for the personal markers factor as 0.93 and 0.94 in the initial and post-hoc CFA models respectively. The composite reliabilities for both latent factors were higher than the minimum required composite reliability of 0.7, which indicated that both latent factors have a high internal consistency suggesting that the loaded measured markers (items) consistently represented the respective identified latent factor [17, 18]. The CFA models showed that both latent factors i.e., the organisational markers and the individual markers have a strong correlation i.e., 0.76 and 0.75 in the initial and post-hoc CFA models respectively. The EFA and CFA results identified and confirmed two significant dimensions i.e., organisational and individual markers of GE in BRCs.

## Discussion

**Fig 4:**
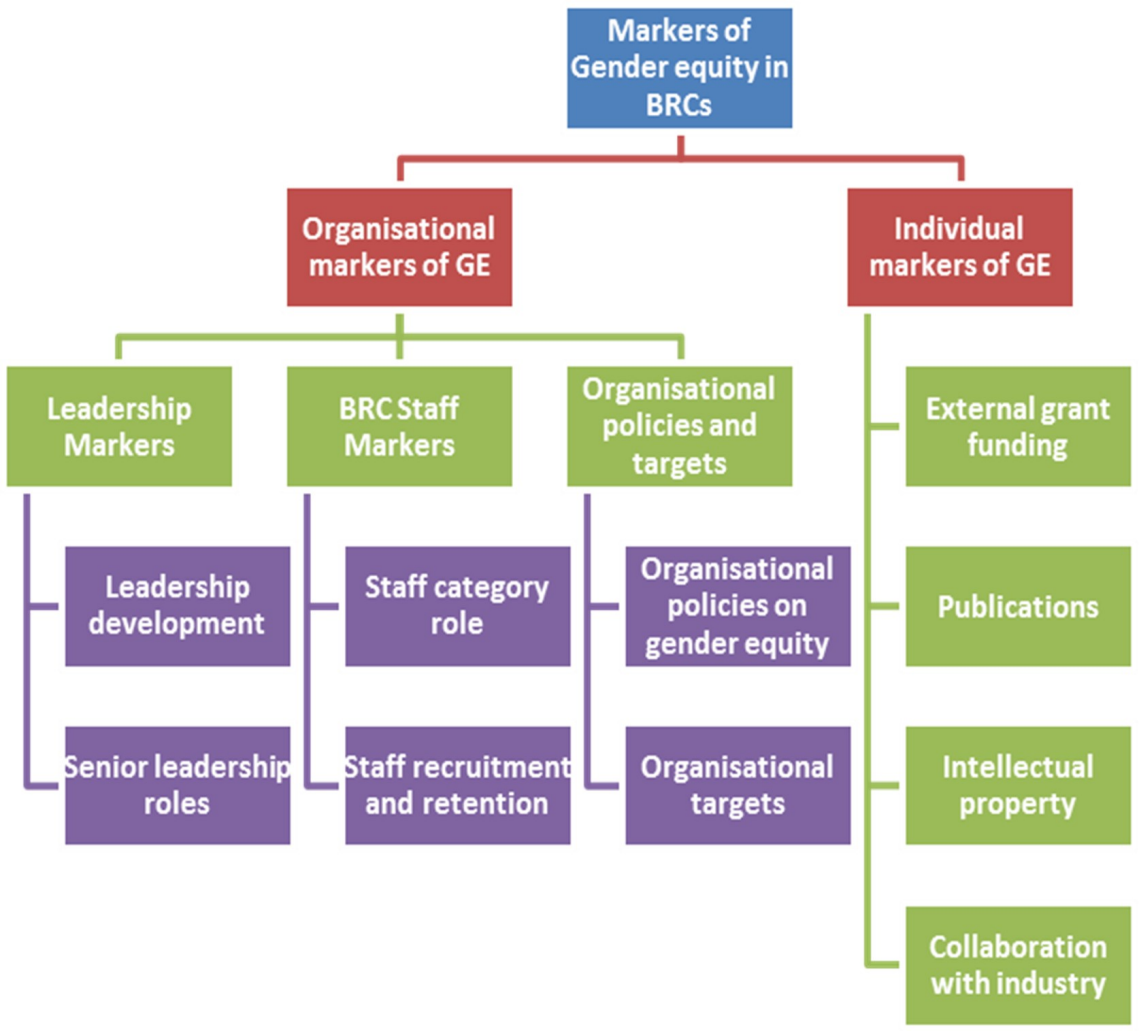
Organisational and individual markers of gender equity in BRCs.

The survey identified a new statistically significant model of GE markers with two distinct dimensions of GE markers (1) organisational markers and (2) individual markers (Fig 4). The present study is the first, to our knowledge, that has developed such a model attuned to the context of NIHR BRCs. Firstly, we discuss the findings in relation to the findings and implications regarding organisational markers, secondly, individual markers.

### Findings and implications regarding organisational markers of gender equity

The dimension of organisational markers of GE identified in our study comprised six markers of GE (Tables 4 and 6, Fig 4). The scrutiny of the wording and content of these six markers by authors showed these six markers could be sub-divided into three sub-dimensions; leadership markers, BRC staff markers and organisational policies and targets markers (Fig 4).

#### Leadership markers

The leadership sub-dimension comprised two markers i.e., leadership development and senior leadership roles. The BRC staff sub-dimension included two markers, i.e. staff category and staff recruitment and retention. While the third sub-dimension of organisational policies and targets encompassed two markers: organisational policies on gender equity and organisational targets (Fig 4).

The high ranking of leadership for GE in this study suggests further local organisational policies may be appropriate. As highlighted elsewhere, local drivers are important to support existing GE initiatives. For example a recent evaluation of Athena SWAN did not indicate a statistical relationship between the Charter and increase in the proportion of female staff over time [10].

#### Organisational policies and targets

Our study provides new potential GE markers within the specific setting of NIHR BRCs. The population is intentionally broader than previous GE research which has predominantly focussed on clinical academic settings [3, 25–31] or Athena SWAN research project populations which focus on university and academic staff [10, 11]. In contrast, this study population includes both NHS staff and clinical and non-clinical university staff at all levels.

### Leadership roles

When scores for all participants were combined, BRC senior leadership roles ranked as the most important marker of GE followed by organisational policies on gender equity (Fig 4) This may reflect findings from a recent evaluation of the Athena SWAN programme which highlighted significant challenges remain in addressing gender balance in the most senior positions in higher education (e.g. professorial, senior management) [9]. It also supports the finding that leadership is a key driver for sustainable organisational change in terms of GE [31].

### Organisational policies on gender equity

Our results suggest that organisational policies on GE are an important measure required. This is typically facilitated at an organisational level by Athena SWAN, but the results suggest more BRC focussed targets may be beneficial at a local level. Linking the direct impact of the introduction of Athena SWAN to the acceleration of GE in an institution is challenging due to the complexity of issues [12].

### Findings and implications for Individual markers of gender equity

The second dimension, i.e. individual markers of gender equity identified included four markers of GE (Tables 4 and 6, Fig 4). The review of the wording and content of these four markers by authors suggested these four markers could be sub-divided into four sub-dimensions, which include research funding, publications, intellectual property and industry collaboration and each of these sub-dimensions included only one GE marker, i.e. marker 6, 8, 9 and 10 respectively (Tables 4 and 6). These findings concur with a recent analysis of lessons learned from the Athena SWAN demonstrates the importance of baseline data for the purposes of benchmarking, and importance of leadership to enable systemic change [12]. At the NIHR Oxford BRC, benchmarking of gender and BRC publications and staff is in place. However, TRO funders may consider encouraging gender benchmarking or making it mandatory. For example, currently the only mandatory request for gender data within BRCs is NIHR academy members (PhD students etc.).

### Markers of achievement in industry and gender equity

Interestingly, collaboration with industry and the Intellectual property emerged as the last and second last very important markers of GE in BRCs reported by 35.4 (n = 86) and 35.8 (n = 87) of participants respectively (Table 2). This may reflect relatively low participation of women in industry [28, 29]. However it is an important area to address given that collaboration with industry is an important metric in BRCs to report to their funder the NIHR.

### Analysis of ranking by gender of participants

Men ranked “leadership development” and “BRC senior leadership roles”, to be most important. Conversely women ranked “organisational policies on gender equity” and “recruitment and retention” to be most important (Table 3). This may reflect women’s perceptions that organisational policies are important drivers of GE and further work is required to support leadership development.

The response rate (36%) in our study is consistent with online questionnaire survey response rates which are typically lower than mail based questionnaires [32] and when participants include clinical professionals [33]. Our results show that the majority of respondents were female suggesting that women were slightly more likely to respond. Research has shown that the relevance of the study topic may impact response rates and so this may have been a factor too [34]. The results also show that women and men rank the importance of the markers of GE differently and a greater proportion of women ranked all markers as the most important compared to the proportion of male participants. However a relatively high proportion of respondents were male (40%) this is key as research has indicated men’s support and perspective is also an important driver of GE in institutions [35].

In regards to the representativeness of the findings in relations to participants who were invited and those who responded, there were no statistically significant differences by participants’ gender and their role in the BRC when Bonferroni corrections were applied.

### Strengths and limitations

To our knowledge, this is the first study to explore views on new markers of achievement for women in academic science specifically in an NIHR BRC setting. Previous research in this field has focussed predominantly on clinical academic settings and Athena SWAN evaluations in Universities [11, 12, 27]. Our study proposes a two-factor model of GE markers in a NIHR BRC setting (Fig 4) based on the conceptual framework derived from the existing literature [14] and views of BRC affiliates across different genders, staff categories, and levels of seniority. Our study compliments the existing literature on gender equity in universities by contributing a context-specific perspective on NIHR BRCs—partnerships between universities and NHS organisations. In doing so, our study contributes to the growing body of literature recognising the complexity of factors producing gender inequality and the importance of context-specific interventions for different categories of staff [36–40] Under the complexity approach, addressing gender inequality requires multiple areas of intervention with a focus on the local context and dynamics [40–42]. Therefore, context-specific GE markers can help to identify areas for improvement, plan interventions, and monitor progress against the goals and strategic objectives for different categories of staff.

Given the significant investment in NIHR BRCs and direct link of demonstrable progress in GE equity, the proposed two-factor model of GE markers is of particular practical relevance to the NIHR Oxford BRC, other NIHR BRCs and policy makers in the UK, and possibly similar translational research organisations in other settings [6]. As a result of this study, the NIHR Oxford BRC has committed to set annual objectives concerning gender equality and monitor progress based on the proposed GE markers in addition to the ongoing support and participation by university department in the Athena SWAN Charter. Moreover, the NIHR Oxford BRC has committed to support new research initiatives on equality, diversity and inclusion [43].

Notwithstanding its strengths, the current study has limitations, which could be usefully addressed in future research. One limitation is that our study is a single-centre study. Given that all 20 NIHR BRCs are structurally similar, future research could establish collaborations across NIHR BRCs to generate large data sets to monitor progress to gender equality on the national level. Gender has been defined as social and cultural constructs associated with being female or male [44]. Due to the relative low numbers we removed the category “self-identify” from the final analysis. Furthermore, we did not collect demographic information to explore intersectional connections between gender, race/ethnicity, and other minority identities. Future research should explicitly take diverse gender identities and intersectional connections with minority identities into account.

Finally, whilst this study did not examine research culture specifically, the importance of the culture of academic medicine for women’s leadership and advancement has been acknowledged in previous research [27, 45]. Future studies should explore cultural issues more in-depth through qualitative research.

## Conclusions

The study has highlighted GE in the workforce is an important indicator for internationally competitive organisations. The findings suggest a two-factor model of markers of achievement for GE with “organisational” and “individual” dimensions. Implementation and sustainability of gender equity requires commitment at senior leadership and organisational policy level. The findings have important implications to inform prospective planning and monitoring within the field of organisational policies and leadership policies to accelerate women's advancement and leadership within the NIHR Oxford BRC, other NIHR BRCs in the UK, and possibly similar translational research organisations in other settings. Enhanced collaborations across NIHR BRCs are suggested to generate large data sets to monitor progress to gender equality at the national level.

## Supporting information

S1 Appendix Survey questionnaire. (DOCX)

S2 Appendix (DOCX)
