# Supplementary material for: Markers of achievement for assessing and monitoring gender equity in a UK National Institute for Health Research Biomedical Research Centre: A two-factor model
Source: PLoS One. 2020 Oct 14;15(10):e0239589. doi: 10.1371/journal.pone.0239589 (PMC7556494; doi:10.1371/journal.pone.0239589)
Supplement: S1 Appendix — (DOCX) [file pone.0239589.s001.docx]

**Markers of achievement for assessing and monitoring gender equity in NIHR Biomedical Research Centres**

**To proceed with the survey, please provide your informed consent below.**

I certify that I am 18 years of age or over. I have read and understood the participant information above. I consent voluntarily to the use of the information I provide in the understanding that it will be aggregated for purposes of analysis so that my responses will not be identified or identifiable.

|  | Yes I consent |
| --- | --- |
|  | No I do not consent |

**1. To what extent do you agree or disagree with the importance of assessing and monitoring gender equity in the following:**

*(For each question, please check the box which best matches your personal view on how important it is to measure the indicators below according to gender e.g. breakdown of number of men and women)*

|  | Very  important | Important | Neutral | Not  important | Not at all  important |
| --- | --- | --- | --- | --- | --- |
| **BRC senior leadership roles**: e.g. Director, Steering Committee Member, Theme leader & Co-lead |  |  |  |  |  |
| **Leadership development**: e.g. Gender-sensitive leadership programmes, succession plans |  |  |  |  |  |
| **BRC staff category**: e.g. Principal Investigator, Researchers, Trainees and Admin & Support staff |  |  |  |  |  |
| **Recruitment & retention**: e.g. Number of Staff recruited and promoted |  |  |  |  |  |
| **BRC funding**: e.g. Distribution by Theme, Gender and Role |  |  |  |  |  |
| **External grant funding**: e.g. Total amount, Role on the grant, Number of grants and Success rate |  |  |  |  |  |
| **Esteem indicators**: e.g. NIHR Senior Investigators, Funding panel membership, Invited plenary speakers, Fellowships of learned societies, Honours and Awards |  |  |  |  |  |
| **Publications**: e.g. Authorship (First / Corresponding / Senior author) and Type of Publication (Journal articles and Conference papers) |  |  |  |  |  |
| **Intellectual property**: e.g. Number of Patents, Licenses and Spinouts |  |  |  |  |  |
| **Collaboration with industry**: e.g. Board membership, Joint grants and Advisory roles (non-executive directorships) |  |  |  |  |  |
| **Patient & public involvement:** e.g.  Representative Number of Men and Women Speakers and Participants |  |  |  |  |  |
| **Organisational policies on gender equity**: e.g. Personal Development Training, Mentoring, Sponsorship and Career Development |  |  |  |  |  |
| **Organisational Targets**: e.g. Creating BRC targets for Gender Equity |  |  |  |  |  |

**2. Please state any other indicators related to gender equity** in your staff category that the BRC should assess and monitor?

**Finally, please tell us about yourself**

**3. Your current role in the NIHR Oxford BRC** *(Pease check one box only)*

|  | Investigator (e.g. PI/COPI/CI) |
| --- | --- |
|  | Research Associate (e.g. Researcher and Research Fellow) |
|  | Admin/Technical/Professional/Support Associate |
|  | Trainee/PhD student |
|  | Prefer not to say |

***4. Duration of working for / affiliation with the NIHR Oxford BRC*** *(Pease check one box only)*

|  | Up to 2 years |
| --- | --- |
|  | 3-7 years |
|  | More than 7 years |
|  | Prefer not to say |

**5. Gender** *(Pease check one box only)*

|  | Male |
| --- | --- |
|  | Female |
|  | Prefer to Self Describe |
|  | Prefer not to say |

**6. Age** *(Pease check one box only)*

|  | 18-30 years |
| --- | --- |
|  | 31-40 years |
|  | 41-50 years |
|  | 51-60 years |
|  | 61+ years |
|  | Prefer not to say |

**7. Please use this box to provide any comments or suggestions on new ways of measuring gender equity in BRCs:**

**This concludes the survey. Thank you very much for your help.**

**For inquiries**, **please contact** **Dr Lorna Henderson** via email:[Lorna.Henderson@ouh.nhs.uk](mailto:Lorna.Henderson@ouh.nhs.uk)
