# Supplementary material for: Markers of achievement for assessing and monitoring gender equity in a UK National Institute for Health Research Biomedical Research Centre: A two-factor model
Source: PLoS One. 2020 Oct 14;15(10):e0239589. doi: 10.1371/journal.pone.0239589 (PMC7556494; doi:10.1371/journal.pone.0239589)
Supplement: S2 Appendix — (DOCX) [file pone.0239589.s002.docx]

**BRC Senior Leadership roles**: absolute and relative numbers of female leaders at all levels, e.g. Director, Steering committee member, Theme leader and co-lead

**Leadership development:** e.g. Gender- sensitive leadership programmes, succession plans

BRC staff category: e.g. Principal Investigator, Researchers, Trainees and Admin & Support staff

**Recruitment and retention:** e.g. absolute and relative numbers of staff recruited and promoted

BRC Funding: e.g. Distribution by Theme, Gender and Role

**External Grant funding:** e.g. Total amount, role on the grant, number of grants and success rate

**Esteem indicators**: e.g. NIHR Senior Investigators, funding panel membership, invited plenary speaker, fellowship of learned societies, honours and awards

**Publications:** e.g. Authorship (First / Corresponding / Senior author) and Type of Publication (Journal articles and Conference papers)

**Intellectual properties**: e.g. Number of patients, licenses and spinouts

**Collaboration with industry:** e.g. Board membership, joint grants and advisory roles (non-executive directorships)

**Patient and public involvement**: e.g. Representative number of men and women speakers and participants

**Organisational policies on Gender equity**: e.g. Personal development training, mentoring, sponsorship and career development

**Organisational targets:** e.g. Creating BRC targets for Gender equity
